# Unusual spread of TB: A case report of hepatic tuberculosis^☆^

**DOI:** 10.1016/j.radcr.2022.06.019

**Published:** 2022-07-08

**Authors:** Made Agatrini Nugia Pramesti, M. Hidayat Surya Atmaja

**Affiliations:** Department of Radiology, Faculty of Medicine Airlangga University, Dr. Soetomo General Hospital Surabaya

**Keywords:** Tuberculosis, Hepatic tuberculosis, Abdominal CT

## Abstract

Tuberculosis (TB) is prevalent in underdeveloped and developing countries, mainly in rural areas, with indistinct clinical manifestations. Lungs are the most affected organs; however, tuberculosis may invade almost all human body systems, including the liver. We provided a case study of a young male adult with hepatic tuberculosis. The diagnosis was established by discovering hypodense nodules with rim enhancement in the liver on CT scan and granulomatous inflammation with caseating necrosis on biopsy. Although having unspecified clinical manifestations, novel liver imaging, combined with pathology examination, is capable of depicting the nature of this entity. Regarding the fact that *Mycobacterium tuberculosis* culturing may be challenging in most settings, imaging plays a vital role in diagnosing, treatment guiding, and patient follow-ups.

## Introduction

Tuberculosis infection counts for a significant proportion of worldwide morbidity and mortality, especially in the tropical climate. In the past few years, the incidence has risen in many developing nations.

Tuberculosis is an airborne disease and has severe outcomes in which *Mycobacterium tuberculosis* is the primary pathogen. The pulmonary organ is the main system to be affected; nonetheless, tuberculosis may affect the other parts of the body. Extrapulmonary tuberculosis made up roughly 15%-20% of all cases, with abdominal TB accounting for less than 1% [3].

**Fig. 1:**
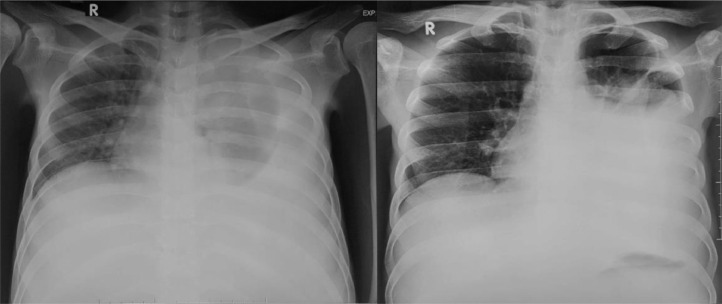
Plain chest radiograph (A) on December 2021 and (B) on January 2022, in which the effusion significantly increased.

**Fig. 2:**
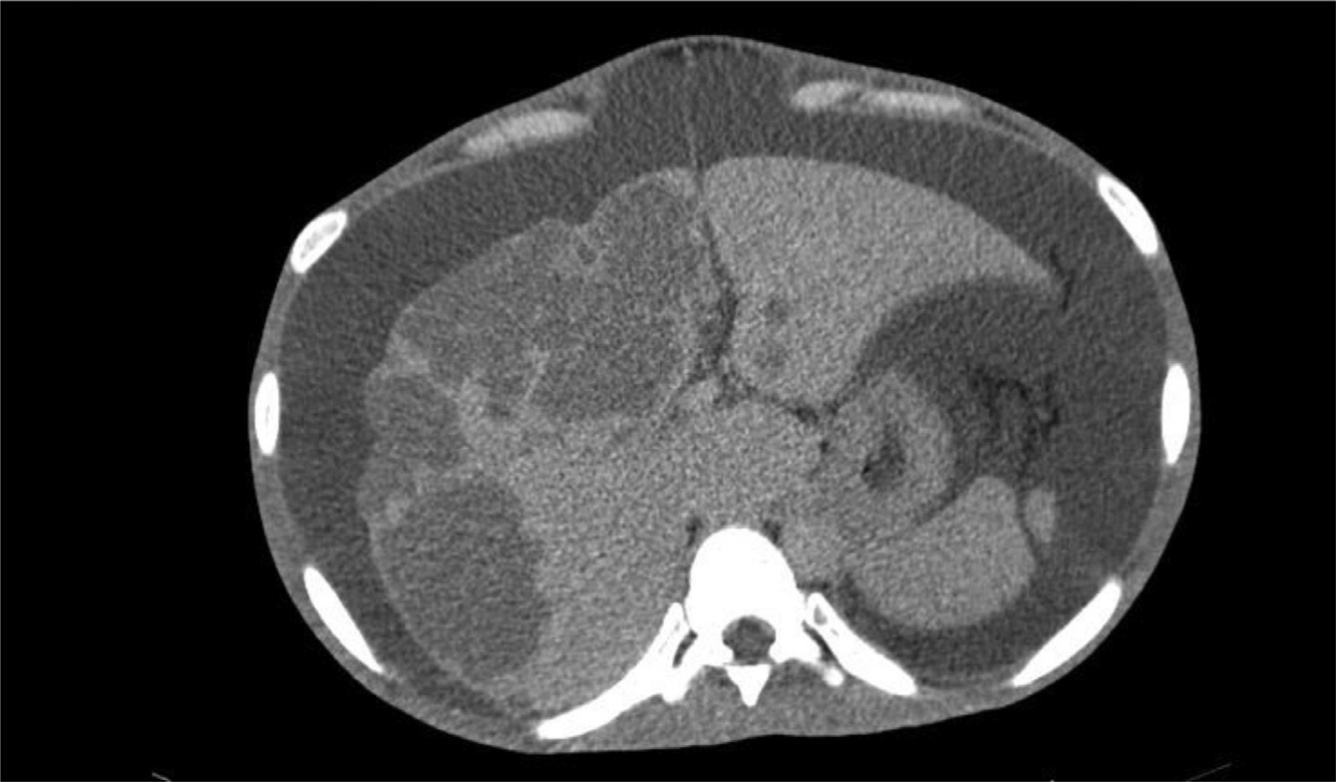
Axial abdominal MSCT illustrated multiple nodules with distinct margins at both the right and left lobes of the liver.

**Fig. 3:**
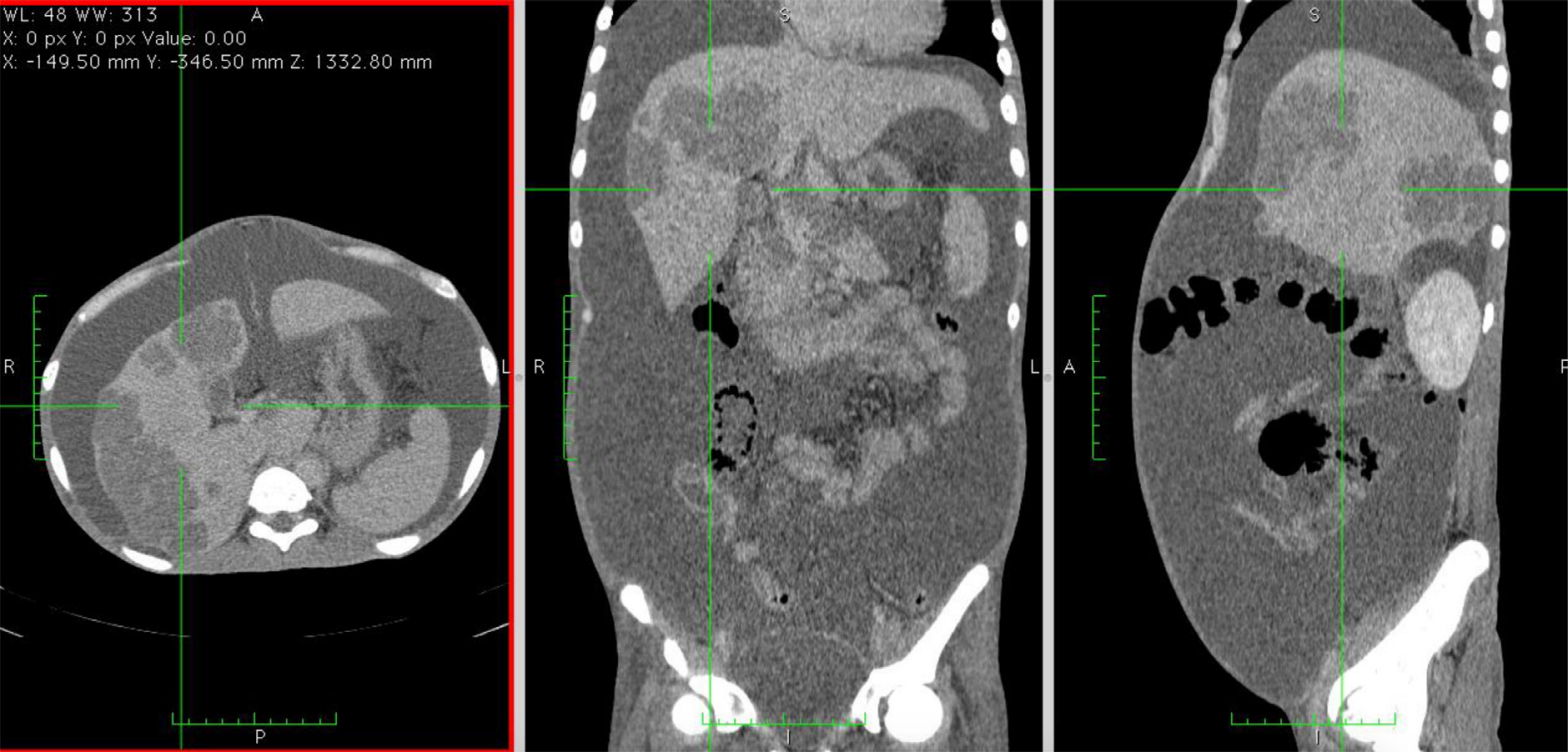
(A) Axial, (B) coronal, and (C) sagittal delayed phase abdominal MSCT depicted multiple nodules with distinct margins at the liver lobes, surrounded by ascites fluid collection within the abdominal and pelvic cavity.

**Fig. 4:**
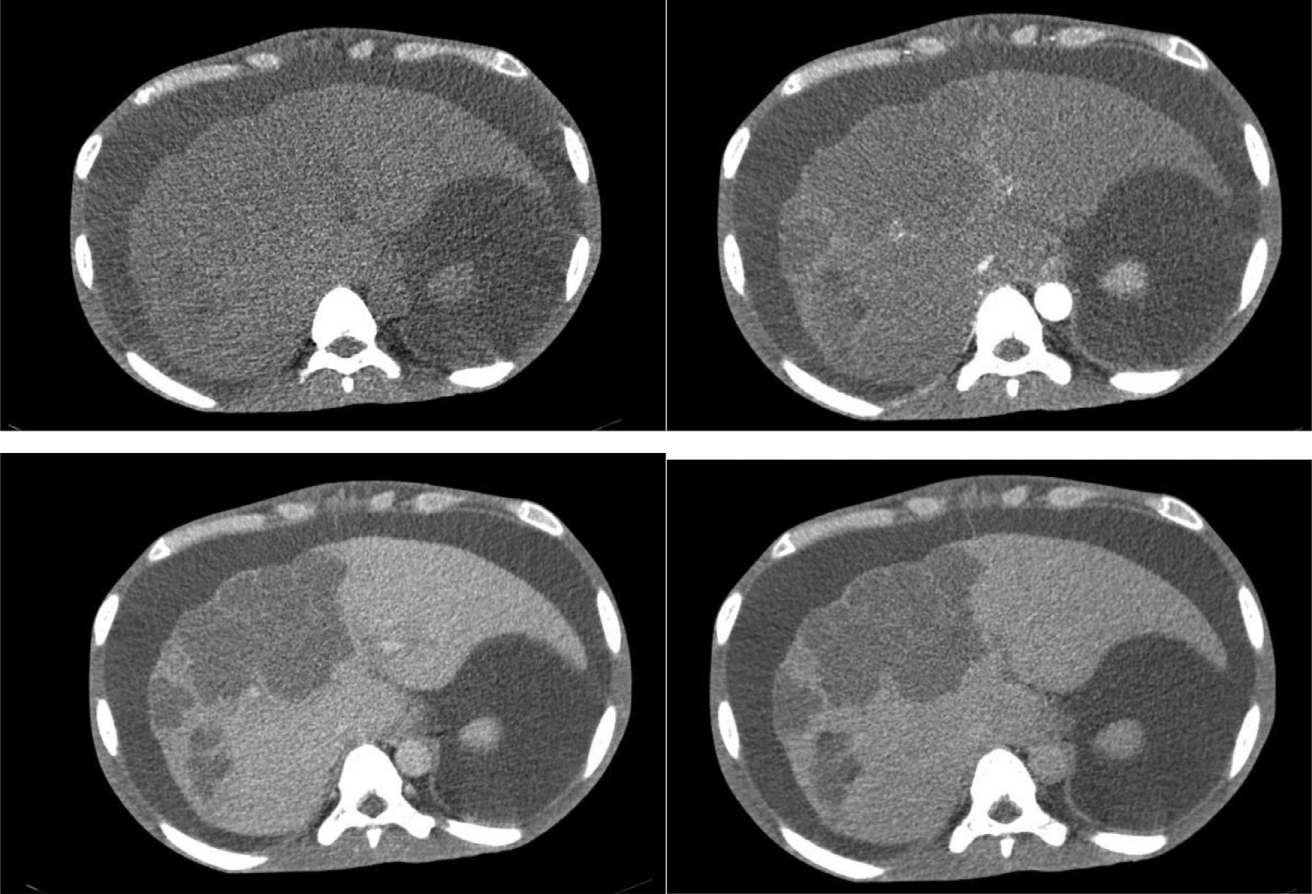
Axial section of abdominal MSCT with contrast administration showed contrast enhancement pattern from (A) noncontrast, (B) artery phase, (C) vein phase, and (D) delayed phase.

One of the rarest entities of tuberculosis is hepatic TB, reported in only a few pieces of literature nowadays, and because of this, no unique characteristics in the CT scan have been described. Internal calcification may lead to the diagnosis; nevertheless, some non-specific diagnostic pictures and pathology confirmation might be requisite [1,4]. However, since these features may sometimes overlap with other diseases, such as benign and malignant masses, they were frequently misinterpreted as uncertain lesions; therefore, it is mandatory to be able to recognize and distinguish between malignant metastatic and TB lesions in the CT scan modality.

**Fig. 5:**
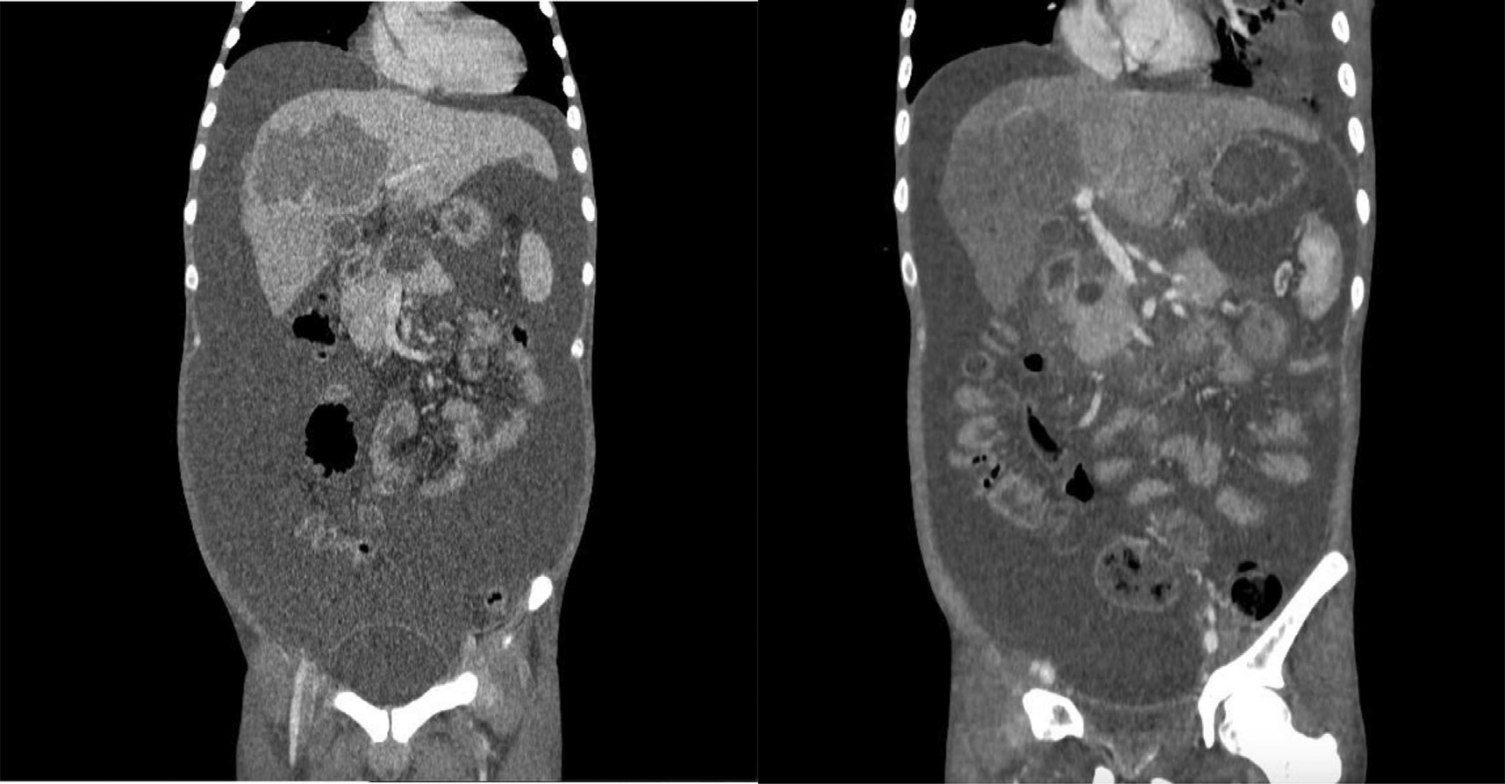
Panel (A) is the liver condition before the course of tuberculosis regimens (November 2021), and panel (B) is the follow-up CT scan after 3 months of treatment (January 2022). Even though the lesions were visible in both pictures, we could appreciate the improvement in the second result.

**Fig. 6:**
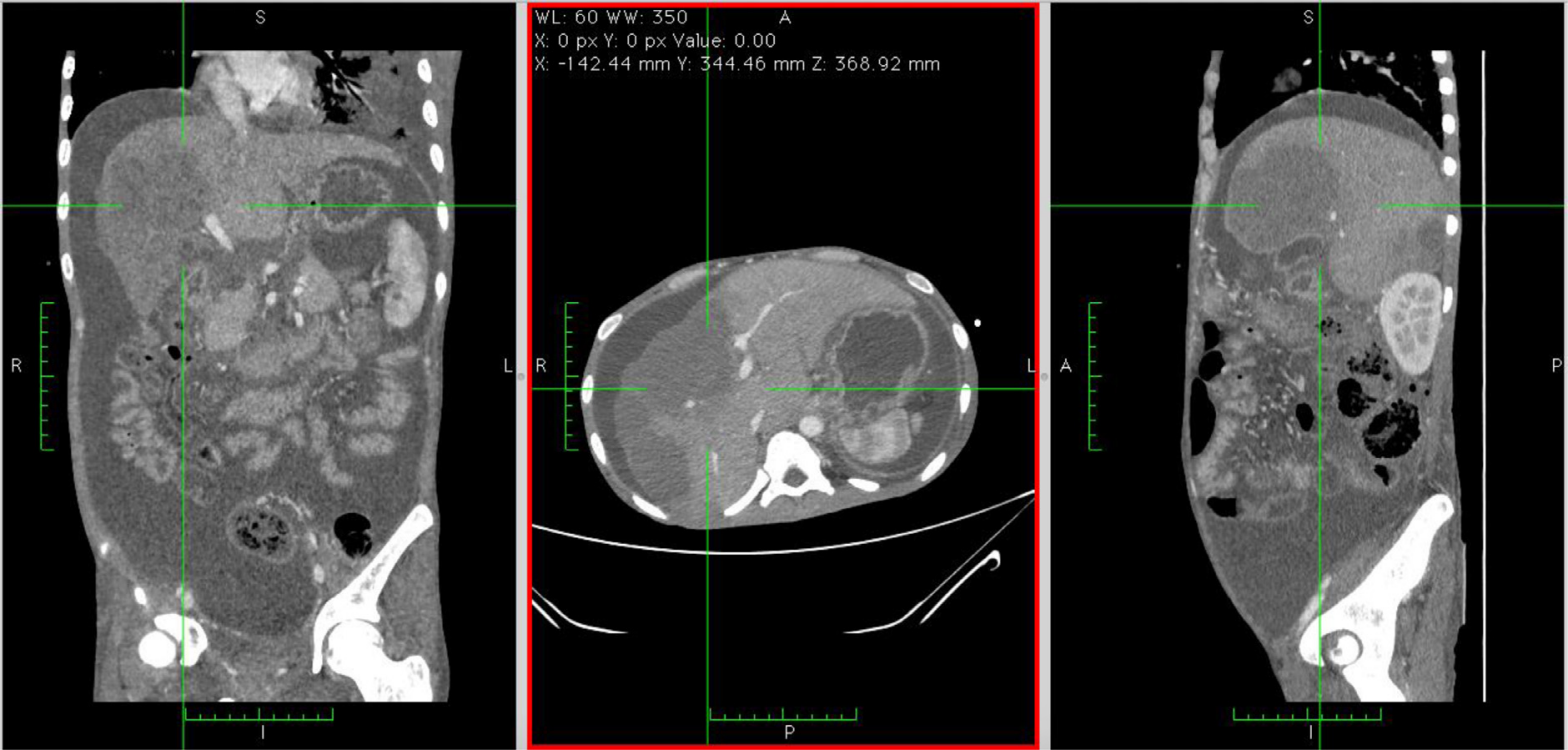
Abdominal CT scan at 3-month follow-ups after the treatment course; the liver underwent a noticeable recovery compared to the first examination.

## Case report

A 25-year-old male patient presented to our center in October 2021 with abdominal pain and enlargement for the past 5-6 months, with the symptoms exaggerated within the last one month. He was unable to sit due to weakness, without nausea or vomiting; nevertheless, he could only eat a spoon every meal. History of 3 weeks of hospitalization was recorded with 14 L of ascites aspirated in total. He also complained of shortness of breath 3 days prior to his hospitalization, and 200 mL of pleural fluid had been aspirated. The patient had experienced these symptoms before the second surge of the COVID-19 wave in the country and had never been vaccinated. Although no history of COVID infection had been reported by him.

During the imaging evaluation, the patient was weak, with no fever, dark stool, bloody vomiting, icterus, and he could defecate daily. He also reported significant weight loss and decreased appetite 4 months prior to the current visit.

Since he was 15 years old, he also had a smoking history and admitted that he could consume 2 packs of cigarettes daily. No account of tuberculosis disease in his family member nor contact with other TB patients had been recorded. He has been unemployed recently.

His laboratory report showed hypoalbuminemia and anemia, with a plain chest radiograph describing left pleural effusion, which was evacuated afterwards.

A triple contrast abdominal CT was also conducted, depicting multiple nodules with distinct margins at both right and left lobes of the liver, followed by core biopsy resulting in granulomatous inflammation and wide necrosis, with no signs of malignancy. In addition, the CT scan image demonstrated ascites collection within the abdominal to the pelvic cavity, with the serial puncture indicating negative for malignancy.

The patient was diagnosed with hepatic tuberculosis and in the intensive phase of tuberculosis treatment, which yielded a satisfying outcome.

## Discussion

Tuberculosis (TB) is defined as a disease affecting the lungs and systemic organs entailed by *M. tuberculosis* infection (MTB), aerobic bacilli bacterium. According to WHO, it remains a primary public health issue, with the estimated prevalence involving around one-third of the global population and new infections roughly 1% of the global population annually. In 2013, it was counted that there were about 9 million cases of novel TB infections and approximately 1.3-1.5 million deaths worldwide [5,6].

Despite the pulmonary system being the primary organ affected by tuberculosis, other systems may be invaded by the pathogen as well, with unclear and vague clinical presentation and radiology features. Hepatic TB is a rare type of extrapulmonary TB, and its incidence has been rising among immunocompromised patients. It occasionally happens in the second to sixth decade of an individual life, with a higher proportion in males [2,[5], [6], [7].

Hepatic tuberculosis rarely happens and comprises less than 1% of all tuberculosis infections. It is uncommon because of the low oxygen pressure in the hepatic tissue, which hinders the growth of aerobic microorganisms. This problem may occur in almost all age groups but more frequently in young adults [7,8].

The ailment may arise as a primary disease itself or emerge from other TB focal sites as a secondary infection. Miliary disease in the liver develops as the tuberculous bacilli reach the organ via the hepatic artery from pulmonary tuberculosis. In several conditions, the pathogen may invade the liver from the portal vein, especially with the gastrointestinal tract involvement [2,3]. In the localized hepatic TB, the portal vein route is the expected course. The bacteria may also reach the liver through the lymphatic system or due to the rupture of the lymph nodes containing the bacilli along the portal tract. Regardless of the entry port, the liver responds to this invasion by forming a granuloma tissue.

In general, this condition might not have a clinical manifestation; instead, it was usually detected accidentally when the patient was being evaluated for vague symptoms. Most cases exhibited nonspecific features, such as abdominal pain, fever, night sweats, weight loss, liver enlargement, epigastric pain, and jaundice. Jaundice has been reported for 35% of cases and is usually an obstructive type. The main characteristic of TB in the CT images was multiple lesions with varying density, indicating several stages of their development. Furthermore, TB diagnosis depends on the existence of epithelioid cell granuloma with caseating necrosis or the acid-resistant bacilli in the aspirated pus or biopsied tissue. In this case study, the parenchymal lesions showed rim enhancement or poor enhancement [9].

In the cross-sectional imaging study, hepatic TB may be comprehensively classified as micronodular and macronodular forms. Micronodular configuration refers to miliary tuberculosis where the lesions measure roughly 0.5-2 mm in diameter, while macronodular fashion may appear as 1-3 cm multiple lesions or a large tumor mass. Such a lump is typically pictured with low attenuation or without peripheral enhancement on CT (hypo or nonenhancing center of the lesion represents the caseating necrosis area, whereas the peripheral edges are similar to the outer granuloma tissue). A low attenuated lesion with center enhancement may be visible as the acute phase of the disease takes course. In addition, a mixed-type hepatic TB has also been described, with micronodular and macronodular features coexistence. In our patient, there was multiple hypodense lesion with ring enhancement on contrast administration [3,7] (Fig. 1, Fig. 2, Fig. 3, Fig. 4, Fig. 5, Fig. 6).

Diagnostic criteria for hepatic tuberculosis are based on clinical history, laboratory tests, imaging features, and pathology examination. Early detection is crucial as it leads to consideration for treatment initiation, avoiding misdiagnosis, and exploratory surgery. Timely recognition in our 4 misdiagnosed cases was able to conduct accurate management with antitubercular chemotherapy and avoid surgery.

However, hepatic TB may be challenging to treat, given the possibility of contralateral lobe reactivation, even though the disease resides in the ipsilateral lobe [2,^8^,9]. Therefore, prompt identification and remedy are indispensable in order to preserve the remaining liver function. It is vital to acquire knowledge and awareness about the broad spectrum of manifestation and discovery because early identification of extrapulmonary TB remains a challenge in many instances. High clinical suspicion is required to recognize this entity for medical management; even so, liver biopsy and pathology investigation provide a definitive diagnosis. Delayed treatment may instigate hepatic failure and death. Often, it is mandatory to explore further organ involvement and inspect for additional coexistent problems such as malignancy.

In the majority of cases, antituberculosis regimens are effective with immediate detection and management, usually yielding a good prognosis. Typical antituberculosis medications (isoniazid, rifampicin, pyrazinamide, and ethambutol) are the recommended protocol.

## Conclusion

Hepatic tuberculosis has unspecified clinical manifestation, and therefore, imaging modality, along with CT-guided fine-needle biopsy, offers an excellent diagnostic value. The existence of hypodense nodule with dilated biliary duct associated with lobar atrophy indicated a consistent imaging feature of hepatic TB in this series, especially with active lung disease. Unless there is a high suspicion of tuberculosis, its diagnosis is often ignored.
